# Identification of Selective BRD9 Inhibitor via Integrated Computational Approach

**DOI:** 10.3390/ijms232113513

**Published:** 2022-11-04

**Authors:** Maria Mushtaq Ali, Sajda Ashraf, Mohammad Nure-e-Alam, Urooj Qureshi, Khalid Mohammed Khan, Zaheer Ul-Haq

**Affiliations:** 1 Dr. Panjwani Center for Molecular Medicine and Drug Research, International Center for Chemical and Biological Sciences, University of Karachi, Karachi 75270, Pakistan; 2 Department of Pharmacognosy, College of Pharmacy, King Saud University, Riyadh 11451, Saudi Arabia; 3 H.E.J. Research Institute of Chemistry, International Center for Chemical and Biological Sciences, University of Karachi, Karachi 75270, Pakistan; 4 Department of Clinical Pharmacy, Institute for Research and Medical Consultations (IRMC), Imam Abdulrahman Bin Faisal University, Dammam 31441, Saudi Arabia

**Keywords:** BRD9, cancer, structure-based pharmacophore, molecular docking, molecular dynamic simulation, MM-GBSA

## Abstract

Bromodomain-containing protein 9 (BRD9), a member of the bromodomain and extra terminal domain (BET) protein family, works as an epigenetic reader. BRD9 has been considered an essential drug target for cancer, inflammatory diseases, and metabolic disorders. Due to its high similarity among other isoforms, no effective treatment of BRD9-associated disorders is available. For the first time, we performed a detailed comparative analysis among BRD9, BRD7, and BRD4. The results indicate that residues His42, Gly43, Ala46, Ala54, Val105, and Leu109 can confer the BRD9 isoform selectivity. The predicted crucial residues were further studied. The pharmacophore model’s features were precisely mapped with some key residues including, Gly43, Phe44, Phe45, Asn100, and Tyr106, all of which play a crucial role in BRD9 inhibition. Docking-based virtual screening was utilized with the consideration of the conserved water network in the binding cavity to identify the potential inhibitors of BRD9. In this workflow, 714 compounds were shortlisted. To attain selectivity, 109 compounds were re-docked to BRD7 for negative selection. Finally, four compounds were selected for molecular dynamics studies. Our studies pave the way for the identification of new compounds and their role in causing noticeable, functional differences in isoforms and between orthologues.

## 1. Introduction

Post-translational modification of histone proteins has a significant contribution to the epigenetic mechanisms. These modifications are dynamic and reversible, coordinated by signaling pathways and directed by several enzymes [1], affecting many nuclear processes, including gene transcription and regulation. The most common PTM is the acetylation of histone proteins, which generally occurs on highly conserved lysine (K) residues. The presence of the acetyl group on histone lowers its net positive charge by one, consequently reducing the electrostatic interaction between negatively charged DNA and positively charged histones, resulting in a more relaxed and transcriptionally active chromatin [2]. A single lysine modification on histone proteins considerably affects cellular homeostasis by regulating various transcription factors, molecular chaperones, and cellular metabolism [3]. It has now been established that histone acetylation modulates gene transcription in response to physiological and environmental factors by manipulating chromatin structure [4]. Besides the manipulation of chromatin structure, the lysine acetylation (Kac) modulates gene transcription via engaging the bromodomains (BRDs) present in bromodomain-containing proteins (BCPs). The BRDs are evolutionarily conserved protein–protein interaction modules [5] often found in a broad range of chromatin and other transcription-associated proteins.

Of all the BRDs, BRD of the BRD9 is a non-BET BCP that has been reported in pathologies of various cancer types, including bladder cancer, lung adenocarcinoma, esophageal carcinoma, ovarian cancer [6], cervical cancer, hepatocellular carcinoma [7], papillary thyroid carcinoma [8], etc., whereas pharmacological inhibition or genetic knockdown of BRD9 proves to be promising in switching the malignant phenotypes. Furthermore, BRD9 has been inseparably associated with inflammation and Type 2 diabetes owing to β-cell dysfunction [9,10].

Research on BRD9 has been accelerated due to its potential role in diseases. In the last few years, since 2015, the chemical probe landscape of the BRD7/9 was very well established with multiple dual BRD7/9 inhibitors. LP99 is the first reported selective BRD7/9 inhibitor, based on a methylquinolinone scaffold, that effectively inhibits the binding of BRD7/9 to acetylated histones in vivo and in vitro [11]. Other BRD9 inhibitors notably, BI-7273 and BI-9564, used to investigate the biological functions of BRD9 in vivo and in vitro, were demonstrated to be non-toxic by fragment-based screening. The compounds were mainly achieved following a structure-guided chemical optimization that led to the introduction of 2-methyl-2,7-naphthyridin-1-one as an anchor region binder [12]. Subsequently, based on the structural design, GSK in collaboration with the University of Strathclyde reported I-BRD9, a thienopyridone derivative. It has been identified as a selective cytochemical probe for BRD9. In addition, I-BRD9 downregulates cancer and immunology-related genes [13]. Another reported BRD9 inhibitors is a ketone “compound 28” developed from a keto-indolizine BAZ2A/B chemical probe; the compound is potent and selective toward BRD9, and BRD7 showed cellular activity at 1 μM in a fluorescence recovery after photobleaching (FRAP) assay [14]. Despite the presence of many inhibitors, none of these inhibitors has reached clinical trials because most of the inhibitors reported to date are pan or dual inhibitors, liable for many off-target effects [15].

Therefore, there is a strong need to design highly selective BRD9 inhibitors to avoid off-target effects associated with inhibition of other homologous proteins.

Considering the therapeutic potential of BRD9, and lack of selective inhibitors due to the structural resemblance of BRD9 with other BCPs, we aimed to design selective inhibitors against BRD9. 

**Figure 1:**
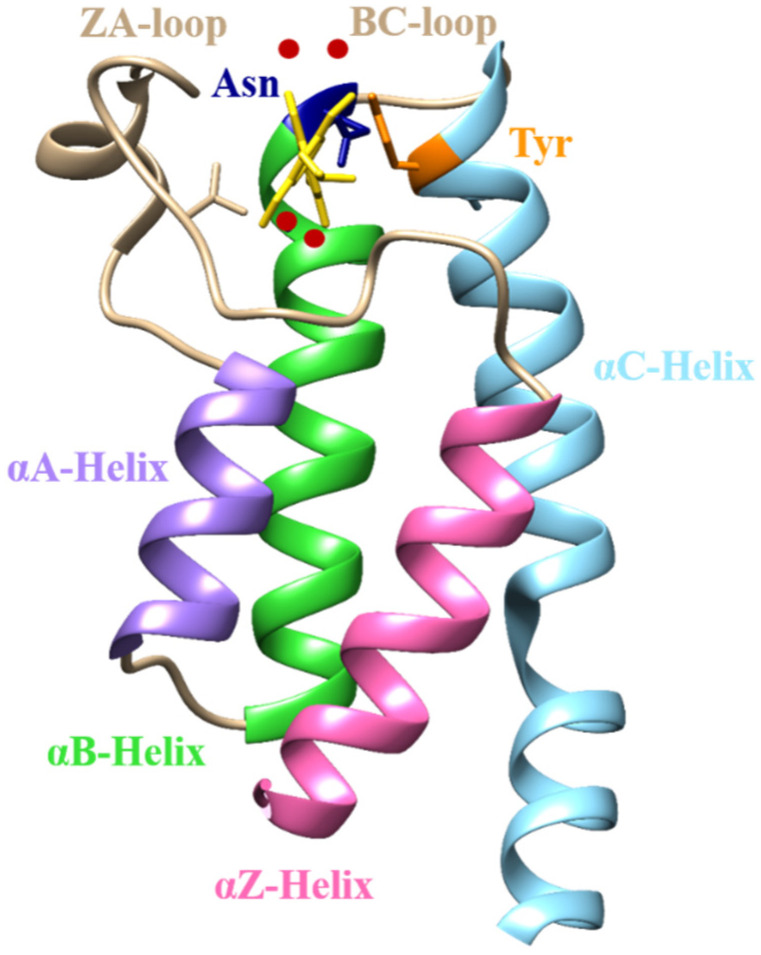
Structure of BRD, rendered through Chimera [17], displaying the four antiparallel alpha helices (αA, αB, αC, αZ) and loop regions (ZA and BC) forming an acetyl binding pocket with conserved water molecules (red sphere).

Bromodomain-containing protein 9 is a subunit of SWItch/Sucrose Non-Fermentable Non-Canonical Bromodomain Associated Factor (SWI/SNF ncBAF), a nucleosome remodeling complex. It has 597 amino acids comprised of two domains, DUF123 and BRD. The BRD of BRD9 is composed of 110 amino acids, assembled into four highly conserved left-handed alpha helices (αZ, αA, αB, αC) linked via two highly flexible loop regions. This arrangement forms a lysine-binding hydrophobic pocket between the ZA and BC loops (Figure 1). Acetyl lysine binding pockets have two conserved residues, Asn (BC loop) and Tyr (ZA loop). The carbonyl group of Asn forms a direct and Tyr forms a water-mediated hydrogen bond (H-bond) with the acetyl group of lysine, playing a crucial role in the recognition of acetyl marks on lysine residues. Another conserved trait observed in the BRDs is the set of water molecules present in the binding pocket. These water molecules serve as an essential feature in virtual screening and designing inhibitors for BRDs as they serve as a bridge between the ligand and binding pocket residues [16].

To investigate the similarities, differences, and binding mode of BRD inhibitors, a comprehensive literature review was carried out along with an exploration of the binding site. The sequence alignment of the three closely related proteins BRD9, BRD7, and BRD4 showed that most residues forming the active sites are located in loops, suggesting that the entry channel is reasonably flexible. By considering those differences, a structure-based pharmacophore model was generated for screening the zinc database to retrieve the potential hits. Designing selective small molecules against BRD of BRD9 offers a powerful tool to comprehend the contributions of this “reader” domain in regulating epigenetic processes. The information revealed by the selective binding of such compounds to BRD9 will certainly resolve many complex mechanisms and their involvement in the regulation of transcription.

## 2. Results and Discussion

### 2.1. Structural Characterization

To achieve improved selectivity, we mainly focused on the crucial and differentiating residues of BRD9. The following differences were observed in the binding site of the isoforms.

**Figure 2:**
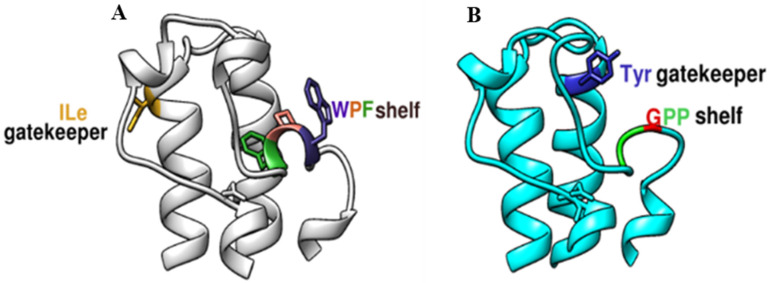
Structural Difference between the binding pocket were retained by Chimera [17], (**A**) BRD4 and (**B**) BRD9, GPP shelf and Tyr106 (gatekeeper) in BRD9 is replaced by WPF shelf and Ile (gatekeeper residue) in BRD4.

In BRD4, adjacent to the BC loop, a WPF shelf composed of Trp, Pro, and Phe is present, while in BRD9, this shelf is made up of Gly and two Phe residues, referred to as the GPP shelf [18] (Figure 2). Moreover, Phe45 in BRD9 is observed to be deflected back to accommodate larger ligands, but this is not the case for Phe83, present at a similar position in the BRD of BRD4 [19]. The other difference is the presence of distinct gatekeeper residues that act as a selectivity filter, present at the starting point of αC helix that was found to interact with the aliphatic region and acetyl group of the acetyl–lysine side-chain, making a wall of the binding pocket [5]. In the case of BRD4, it is Ile, which Tyr replaces in BRD9. As a result, BRD9 has a slightly larger pocket in contrast to BRD4, where the pocket size is restricted by the β-chain of Ile.

**Figure 3:**
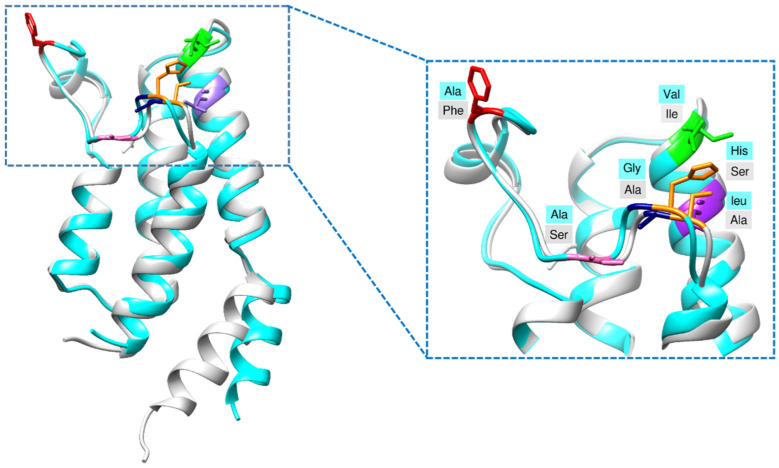
Differences between the acetyl binding pocket of BRD9 (cyan) and BRD7 (gray) were rendered through Chimera [17].

BRD7 is a paralog of BRD9 with an overall structure similarity of 36% [20] and 85% in their BRDs [13]. In the present study, the RMSD of the binding pocket was found to be 0.77 Å. To characterize the binding pockets, co-crystal structures of BRD9 and BRD7 were studied. Although there is high sequence similarity, some differentiating residues are present in the binding site (Figure 3), identified by superposition and some experimental investigations [13,21]. These differences were mainly focused on during pharmacophore modeling and docking-based virtual screening.

### 2.2. Pharmacophore Modeling

**Figure 4:**
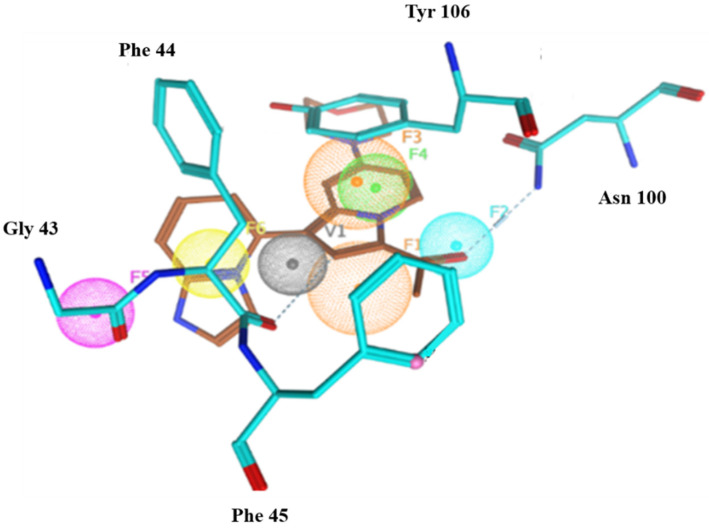
The selected structure-based pharmacophore model generated via MOE 2018 [22] composed of six key features involving donor and acceptor features towards Gly and Asn, respectively, a hydrophobic feature near Tyr106 and two aromatic features near Phe44 and Tyr106.

The pharmacophore model was created by using four co-crystallized structures of BRD9 with known inhibitors, BI-7273, I-BRD9, LP99, and Indolizine-28 (PDB ID; 5EU1 [12], 4UIW [13], 5IGN [11] and 5E9V [14], respectively. Initially, H-bonds, cation–π interactions, π–π interactions and water bridging of the reported structures with His42, Gly43, Phe44 Phe45, Asn100 and Tyr106 were observed in the binding site of BRD9. We also highlighted the differences in the binding site of BRD9 and its isoform, BRD7. Based on crucial and differentiating residues, a pharmacophore model was generated, comprising two aromatic (F1 and F3),one hydrophobic (F4), one H-bond acceptor (HBA) (F2), one H-bond donor (HBD) (F5), and one hydrophobic-aromatic feature (F6). The pharmacophore model’s features were precisely mapped with key residues such as, Asn100, Phe44, Tyr106, and Gly43, all of which play important roles in BRD9 inhibition. In particular, the HBD feature was mapped on the Gly43, which is selectively present in the BRD9 pocket, while the HBA feature was mapped onto the conserved Asn100 residue. The two aromatic features with the projected point were Tyr106, Phe44 and Phe45. A single exclusion volume was also applied to direct the inaccessible areas for any potential ligand and increase steric selectivity (Figure 4).

### 2.3. Statistical Validation

To assess the discriminating power of the pharmacophore model between true positive and true negative, a test set of 13, 041 compounds including (i) active (<500 nM) and inactive (>500 nM) compounds, (iii) BRD4 inhibitors with experimental activity values [11,12,13,14] and a decoy dataset, was utilized.

The hit rate is given in Table 1.

The pharmacophore model was validated by multiple statistical parameters, including sensitivity (Se), specificity and area under curve (AUC). 

Sensitivity is the rate of the true positive compounds retrieved from the database.

Se = T_p_/T_p_ + F_n_ (1)

F_n_ is the number of false negatives, and T_p_ is the total number of actives.

Specificity denotes the percentage of rejected inactives by the particular virtual screening workflow.

Sp = T_n_/T_n_ + F_n_ (2)

T_n_ is the number of true negatives; F_p_ is the number of false positives.

**Figure 5:**
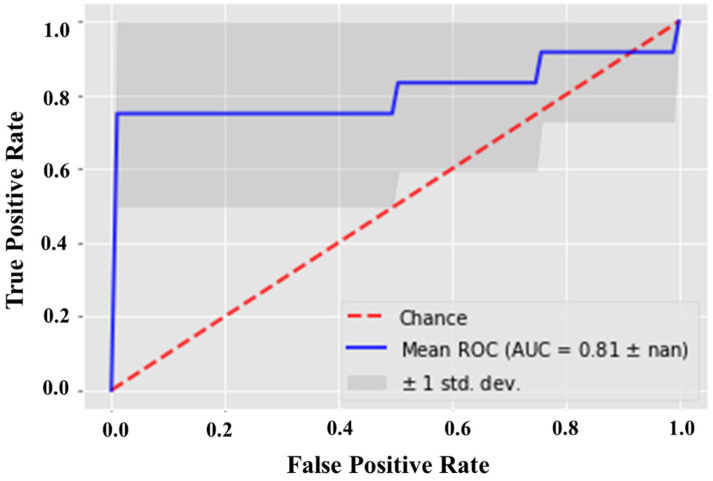
ROC curve validation by using 17 actives and 12,991 decoys, highlighting the efficiency of the modeled structure-based pharmacophore to reliably differentiate between true positives and false positives.

A ROC curve was plotted by setting the score of the active molecule as the first threshold. In a ROC curve, the values lie in the range of 0–1. The curve being closer to 1 or a left-handed hyperbolic indicates an accurate ranking, representing the systematic distribution of actives. Considering the above criteria, the generated pharmacophore model with an AUC of 0.81 (Figure 5) was considered a reliable model for virtual screening.

### 2.4. Pharmacophore-Based Virtual Screening

After validating the pharmacophore model, it was used as a 3D query for the virtual screening of four ZINC database subsets (Section 3.2.1). Overall, 714 compounds were recognized as lead compounds and subsequently subjected to molecular docking studies to refine the results. Screening results are mentioned in Table 2.

### 2.5. Docking-Based Virtual Screening

After benchmarking, docking and interaction analyses of screened compounds were performed to select the BRD9 from the pharmacophore-based virtual screening result. The acetyl binding site of BRD9 is predominantly composed of His42, Phe44, Phe45, Phe47, Val49, Ile53, Asn100, and Tyr106. BRD9 holds a hydrophobic active site; therefore, the hydrophobic interactions cannot be ignored as they play a part in the positioning of the ligand in the active site. Among the active class of inhibitors, BI-7273 was selected as a reference compound as it shows 3-fold and 50-fold more selectivity towards BRD9 as compared to BRD7 and BRD4, respectively.

**Figure 6:**
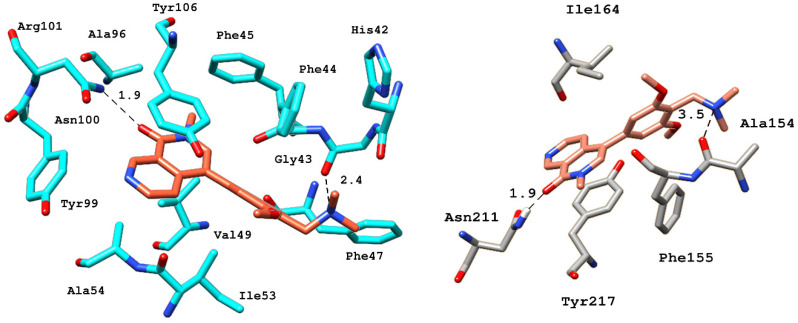
3D representation of various electrostatic and hydrophobic interactions of the reference compound with BRD9 (cyan) and BRD7 (gray) binding pocket residues.

In terms of interaction patterns, π–π stacking was observed with Tyr106 in the BRD9 anchor region. The ligand was further stabilized by a π interaction with Ile53, and T-stacking with Phe44 in the acetyl binding site. A hydrogen bond was observed between the carboxyl groups at the 8th position of the naphthyridinone rings with the carbonyl side chain of Asn100 at a distance of 1.9 Å. The dimethoxyphenyl ring of BI-7273 formed another H-bond with the amine group of Gly43 at a 2.4 Å distance (Figure 6).

**Scheme 1:**
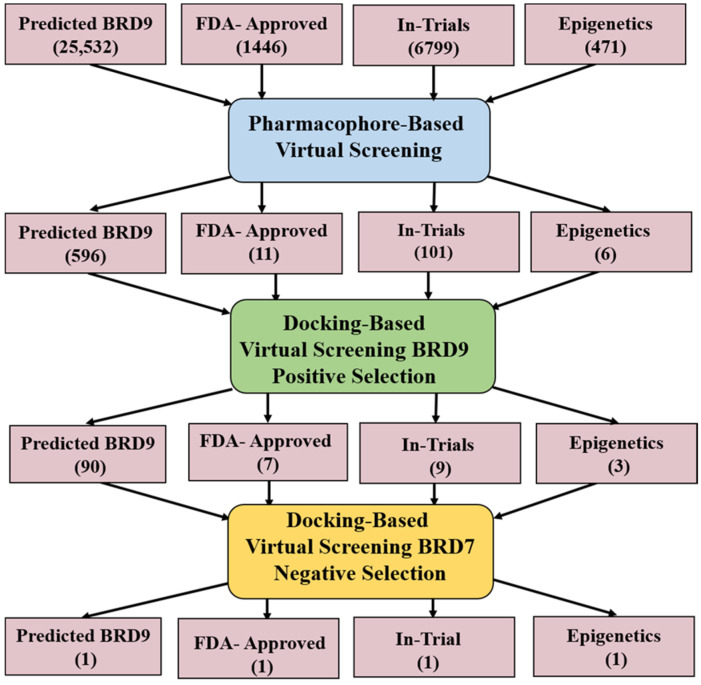
Work flow towards selective BRD9 inhibitors.

To achieve selectivity for BRD9, a scaffold could form interactions with selective residues including Gly43, His42, and Ala54 while occupying the hydrophobic pocket, defined by the Phe44, Phe45, and gatekeeper Tyr106. By considering the above criteria, after binding mode analysis and protein–ligand interaction fingerprints (PLIF) [23] in MOE, 109 compounds shortlisted from all the four databases were further docked to BRD7. This decisive step allowed us to classify the compounds into two groups demonstrating favorable interactions with the binding pocket of BRD7 or BRD9 (Scheme 1). Finally, we ended up with four compounds (Table 3) that showed good interactions with the crucial residues of BRD9 only.

**Figure 7:**
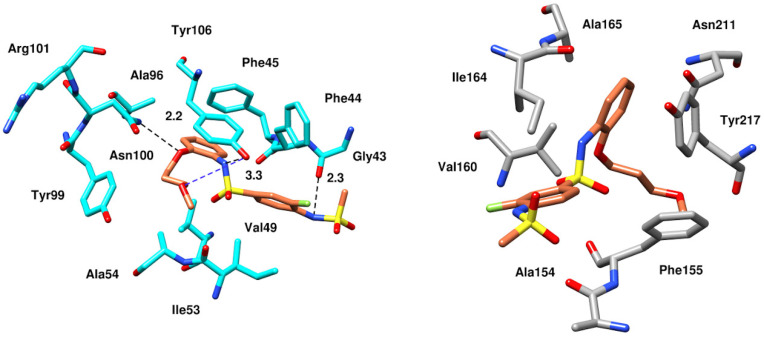
Molecular docking interactions of the binding pockets residues of BRD9 (cyan) and BRD7 (gray) with ZINC433599781 [17].

After PLIF analysis, all four compounds were visually examined to observe their binding modes. Since the binding pocket is hydrophobic, all the compounds exhibited multiple hydrophobic and π–π interactions. Molecular visualization of the BRD9–ZINC433599781 complex revealed that the thioamide group of 2-fluorobenzene interacted with carbonyl from the amide group of selective Gly43 by forming an H-bond at a distance of 2.3 Å. The compound was further stabilized by an H-bond with Tyr106 and conserved Asn100 through its thioamide group at a distance of 3.3 Å and the oxygen of carboxyl adjacent to methoxypropoxy phenyl group at 2.2 Å. Moreover, ZINC433599781 was further stabilized by multiple hydrophobic interactions with Phe44, Phe45, Val49, Ala54, Ala96, Tyr99, and Tyr106, as well as by water bridging with Tyr106 (Figure 7).

**Figure 8:**
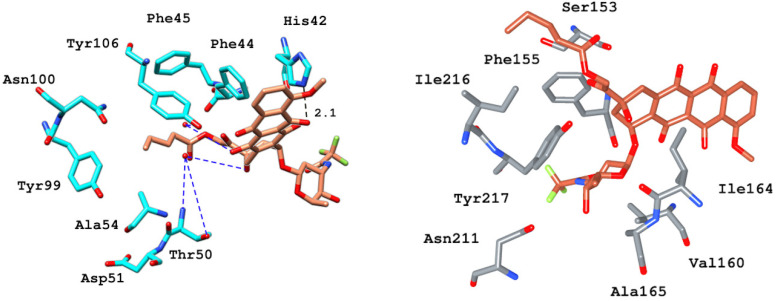
Binding and interaction pattern of the ZINC28232750 in the acetyl–lysine pocket of BRD9 (cyan) and BRD7 (gray) [17].

The binding mode analysis of ZINC28232750, ZINC2036848, and ZINC95589781 with BRD9 revealed that they all fit well into the protein’s binding site. ZINC28232750, an FDA-approved drug, binds in the active site of BRD9 (Figure 8) by forming an H-bond with selective His42 at a distance of 2.1 Å through the carbonyl oxygen of the anthracene ring present at the sixth position. Additionally, the compound is occupied in the pocket by hydrophobic interactions with Phe44, Val49, Thr50, Ile53, Ala54, Tyr99, and conserved Tyr106. Besides these interactions, another important factor contributing to ligand stability is water bridging with Tyr106 and Thr50.

**Figure 9:**
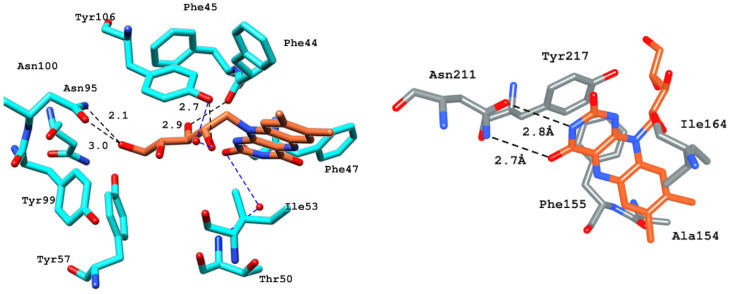
Molecular docking interactions between ZINC2036848 and the binding site of BRD9 (cyan) and BRD7 [17].

The binding of ZINC2036848 in the active site was attributed to H-bonds from its hydroxyl groups to Phe44 and Tyr106 with the tetrahydropentyl moiety at 2.9 Å and 2.7 Å distances, respectively. The pteridine ring established an H-bond with Phe47, and the terminal hydroxyl group of the tetrahydropentyl moiety at the fifth position formed two H-bonds with the amide group of Asn100 at 2.1 and 3.0 Å distance. The complex formation was further derived by the hydrophobic interactions with Phe44 and Ile53 (Figure 9). Similar to the FDA-approved drug, ZINC2036848 was also stabilized by water bridging with Thr50 and Tyr106.

**Figure 10:**
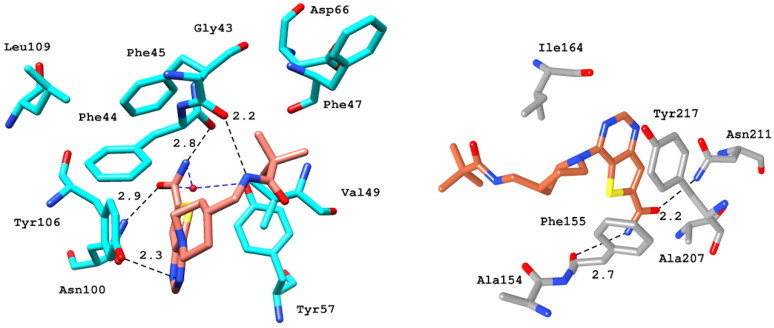
Docked poses of BRD9 (cyan) and BRD7 (gray) with ZINC95589781 [17].

The binding of ZINC95589781, which can be seen in Figure 10, indicates that it has formed multiple H-bonds with active site residues. The amine group of the methypivalamide moiety attached to the piperidine ring formed an H-bond with selective Gly43 at 2.2 Å distance. The amine and carboxyl groups of the carboxamide were involved in H-bond interactions with Phe44 and conserved Asn100 at a distance of 2.8 and 2.3 Å, respectively. The side-chain hydroxyl group of conserved Tyr106 established an H-bond at a distance of 2.9 Å with the nitrogen atom of the pyrimidine moiety. Similar to other compounds, ZINC95589781 was also stabilized by hydrophobic interactions with Phe44, Phe47, and Tyr106, as well as by water bridges with Tyr57.

### 2.6. Molecular Dynamic (MD) Simulation

To acquire detailed information of the complex regarding the stability and the dynamic behavior of the molecular interactions of the top four compounds inside the binding pocket, an MD simulation was performed. In this regard, different parameters such as RMSD, RMSF, RoG and interaction profile were analyzed.

#### 2.6.1. Root Mean Square Deviation (RMSD)

The root mean square deviation (RMSD) is one of the key parameters used to probe the dynamic stability of the complex by evaluating the deviation of protein backbone atoms with respect to time.

**Figure 11:**
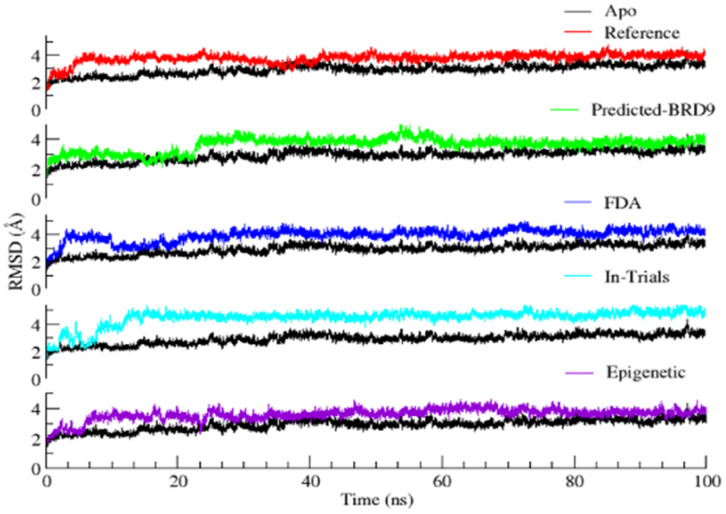
Root mean square deviation (RMSD) of backbone atoms in BRD9 relative to the corresponding Apo (black) and reference compound (red); ZINC433599781 ZINC28232750 (blue); ZINC2036848 (cyan); ZINC95589781 (purple).

The RMSD of the backbone atoms of the protein revealed that the Apo-form of BRD9 was stable with an average RMSD value of 2.9 ± 0.3 Å (Figure 11). On the other hand, the resulting complexes fluctuated slightly for 10 ns. These variations appear to be related, primarily with the release of minor unfavorable ligand–protein steric interactions. After 10 ns, the trajectory frames show a plateau in the RMSD graph, indicating the attainment of the equilibration state of all the complex structures. It states that all complexes adopt a stable binding mode and exhibit relatively small, infrequent fluctuations during the course of the MD trajectory. The average RMSD for the reference compounds, ZINC433599781, ZINC28232750, ZINC2036848, and ZINC95589781, was found to be 3.7 ± 0.3, 3.6 ± 0.4, 3.9 ± 0.4, 4.4 ± 0.5, 3.6 ± 0.3 Å, respectively. The deviation was higher as compared to the Apo form. It might be attributable to the binding of the inhibitor, which increases the conformational flexibility of the binding pocket formed by the two loop regions (ZA and BC), which underwent large changes. The detailed structural differences of the system before and after MD simulation were examined. It was observed that the loop is rearranged and tends to open up a little bit during the course of 100 ns MD simulation to accommodate the ligand inside the pocket. The obtained results suggested that ZINC433599781 and ZINC95589781 were more stable and exhibited fewer fluctuations throughout the simulation period. The overall RMSD profiles suggested that all the complexes were stable and that the results are reliable for further studies.

#### 2.6.2. Root Mean Square Fluctuations

Root mean square fluctuation (RMSF) is one of the key parameters used to probe the mean fluctuations of the dynamic system residues/atoms.

**Figure 12:**
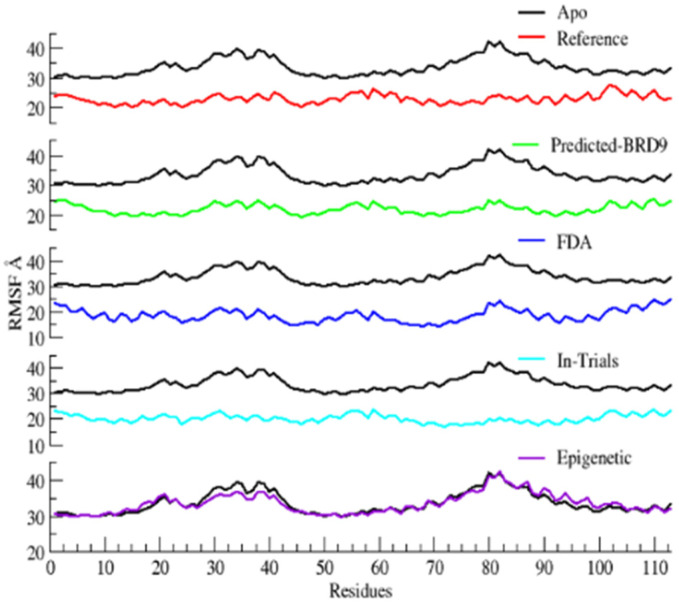
RMSF plots for BRD9 protein with Apo (black) and reference compound (red); ZINC433599781 ZINC28232750 (blue); ZINC2036848 (cyan); ZINC95589781 (purple).

It represents flexibility in reference to the average position of residues in the protein–ligand complexes over time. As expected, the protein with the alpha helix was observed to experience fewer fluctuations than the loop domains. The RMSF graph (Figure 12) of ZINC95589781 exhibited similar fluctuation patterns to Apo, indicating that it does not significantly influence the backbone of the protein.

**Figure 13:**
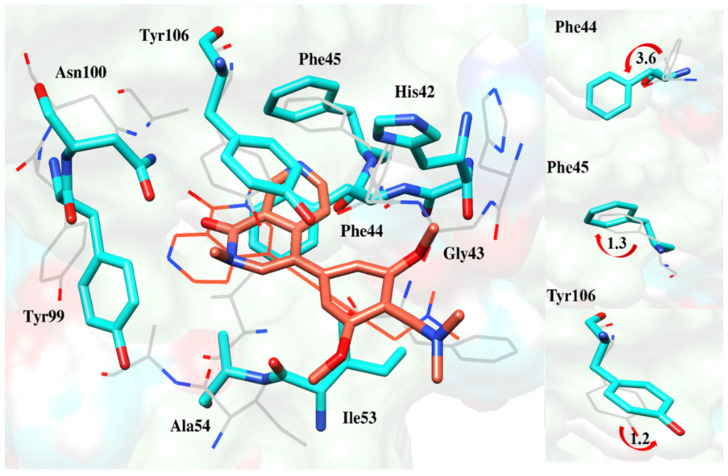
Deflection of Phe44, Phe45 and Tyr106 to accommodate the reference compound deeper inside the pocket [17].

**Figure 14:**
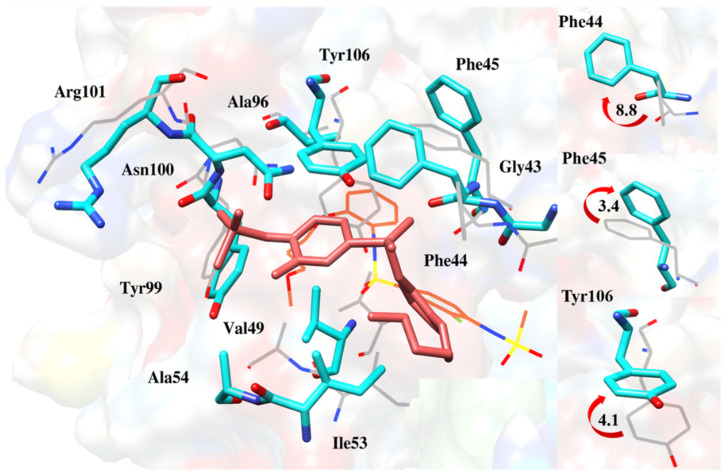
Comparison of docking and MD-averaged poses of BRD9-ZINC433599781 to highlight the deflection of Phe44, Phe45 and Tyr106 [17].

**Figure 15:**
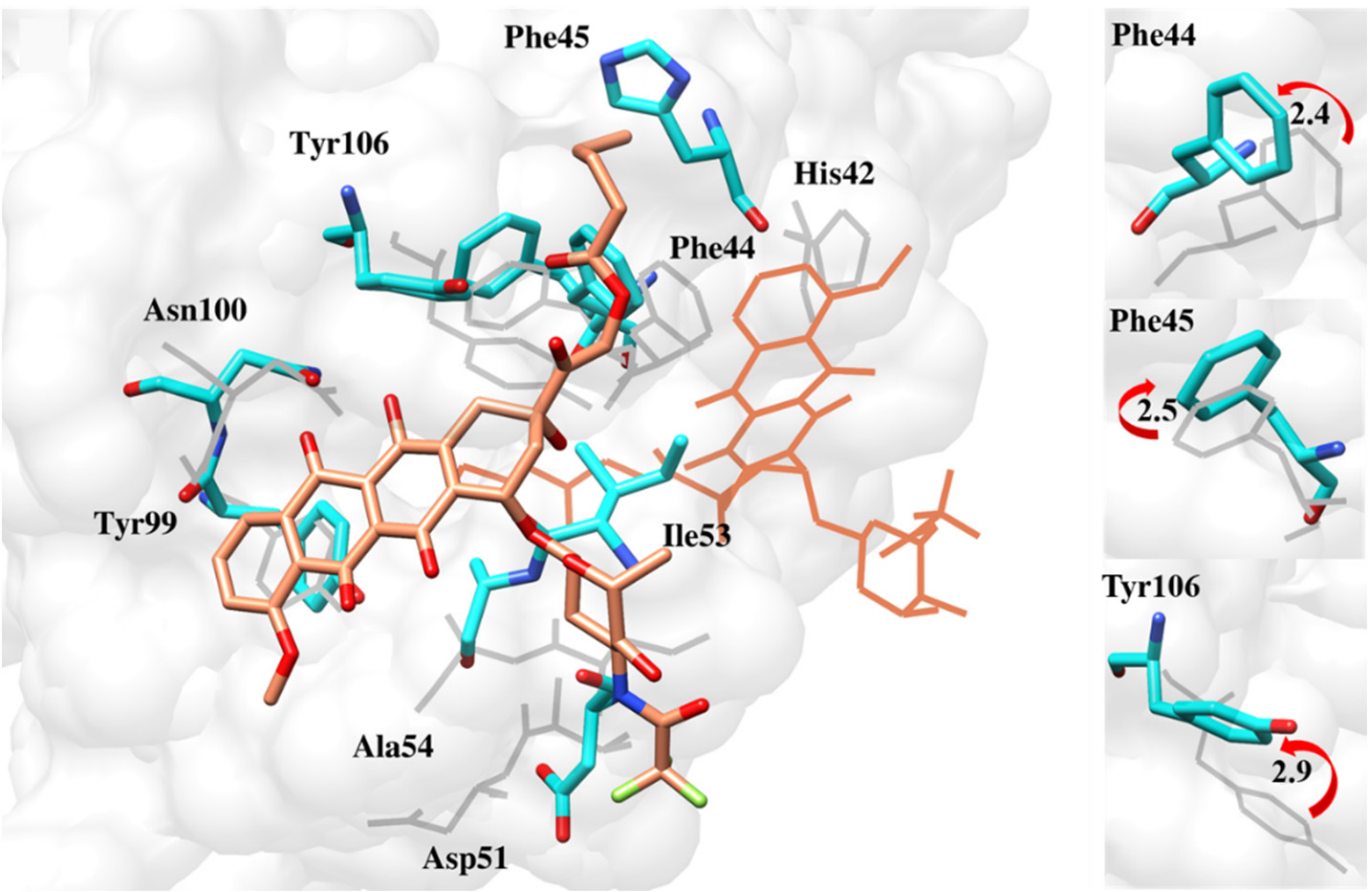
Deflection of Phe44, Phe45 and Tyr106 during MD simulation to accommodate ZINC28232750 deeper inside the pocket [17].

**Figure 16:**
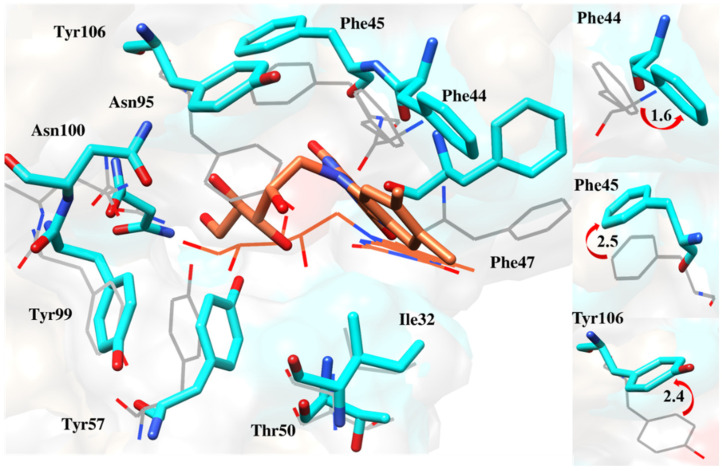
Orientation of Phe44, Phe45 and Tyr106 from MD simulation as compared to docking simulations [17].

**Figure 17:**
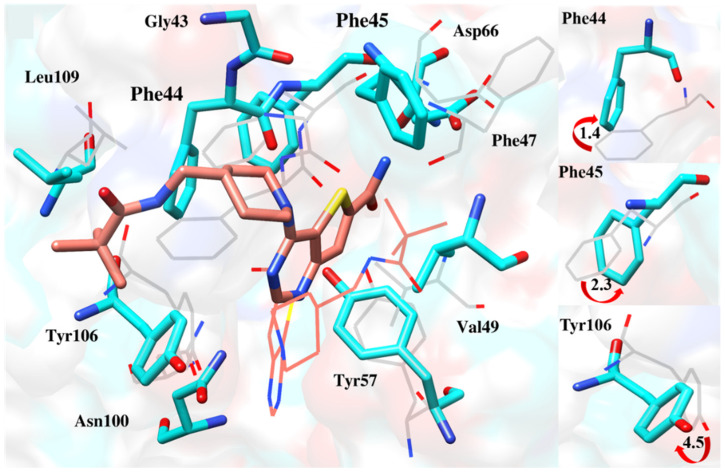
Deflection of Phe44, Phe45 and Tyr106 to accommodate ZINC95589781 deeper inside the pocket [17].

Finding the reason behind the fluctuation pattern, it was worthwhile to observe the interaction of the cavity’s key residue. All the compounds changed their orientations to get inside the cavity, resulting in the fluctuation of active site residues. These include Phe44, Phe45 and the gatekeeper residue Tyr106, which deflected from their original positions to accommodate the ligands (Figure 13, Figure 14, Figure 15, Figure 16 and Figure 17).

#### 2.6.3. Radius of Gyration

The radius of gyration illustrates the size and the compactness of proteins by measuring the distribution of atoms around its axis. The lowest RoG represents the tightest packing, characteristic of α-helices and β-sheets of the proteins. In contrast, loop regions of proteins correspond to a larger value of RoG.

**Figure 18:**
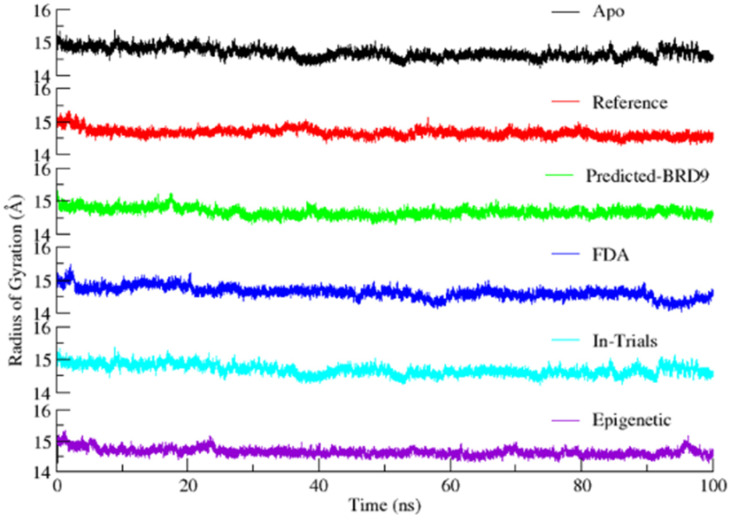
Radius of gyration plots of BRD9, Apo (black); Reference (red); ZINC433599781; ZINC28223750 (blue); ZINC2036848 (cyan); ZINC95589781 (purple).

The trend in RoG of the protein backbone of each MD simulation is shown in Figure 18. The decrease in average values of RoG from 15.5 to 14.6 Å for all the systems during 100 ns MD simulation proposes that all of the systems attained a well-converged state with time. In addition, it also suggested the conformational transition of the protein into a more structured form when the ligand stably occupied the binding site. The stable conformation achieved by the target protein after binding to the compounds is attributed to hydrogen bonds and hydrophobic interactions.

#### 2.6.4. Interaction Pattern of Ligand–Protein Complexes

**Figure 19:**
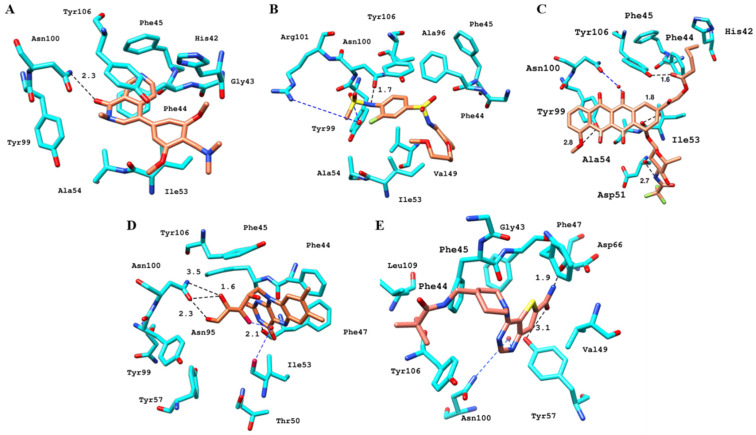
Post-MD interactions pattern of BRD9 (cyan) with (**A**) reference, (**B**) ZINC433599781, (**C**) ZINC282232750, (**D**) ZINC2036848, and (**E**) ZINC95589781 [17].

The simulated binding mode of the reference compound is depicted in Figure 19A. The result indicated the formation of H-bonds between Asn100 and the carboxyl group of the naphthyridine ring. Interestingly, the same bond was also observed in the docking pose and remained persistent within the range of H-bonds, but the distance increased from 1.9 to 2.3 Å. On the other hand, due to the movement of the ligand deeper inside the pocket, H-bonds with Gly43 and π–π-interactions with Tyr106 did not persist. Similarly, the H-bond with His42 has been replaced by π–π interactions with Phe44. Besides this, the compound was stabilized by Ile53 and Tyr106 through hydrophobic interactions (Figure 19A). Overall, no drastic change was observed in the reference compound’s pre- and post-MD conformation and the binding pocket.

On the other hand, all four inhibitors underwent significant conformational changes upon binding to BRD9. In-depth analysis of the KAc sites in the pre- and post-MD states indicated significant movement of Tyr106, Phe44 and Phe45 to accommodate the ligand in the binding site. The superposition of the two states revealed very different positionings of the inhibitor in BRD9.

During MD simulation, some new inter-molecular interactions were formed between ZINC433599781 and BRD9, predominantly responsible for forming a stable complex (Figure 19B). We found that the amide group attached to 2-fluorophenyl forms an H-bond with Asn100 at a distance of 1.7 Å, initially bonded to Gly43. However, Ile53, Tyr106, and Arg101 were involved in hydrophobic interactions, π-stacking and water bridging, respectively. This change in binding mode was due to the flipping of the compound inside the binding pocket. Initially, the aromatic ring of fluorobenzene sulfonamide was in the vicinity of Gly43, which was switched towards Arg101 during MD.

ZINC28232750 is an FDA-approved drug selected for MD simulation on the basis of H-bond interactions with the selective residue His42 of the target protein. During trajectory analysis, it was observed that compound flipped its orientation to 90° and moved inside the binding pocket. As a result, primary interactions were replaced by new ones and multiple H-bonds stabilized the compound. The amide group of ZINC28232750 formed H-bonds with the side chain hydroxyl group of Asp51 at a distance of 2.7 Å. The other H-bond was formed between Ile53 and the carboxyl moiety of the compound with a distance of 1.8 Å. Similarly, methoxy and RCOOR of the compound were involved in an H-bond with the hydroxyl group of Tyr99 and Tyr106 at a distance of 2.8 Å and 1.6 Å, respectively. Moreover, due to the drift of the compound inside the pocket, Asn100 was able to form a water bridge with the hydroxyl group of the drug. In addition to this, the compound was also observed to form multiple hydrophobic interactions with Phe44, Phe45 and Tyr99 (Figure 19C).

ZINC2036848 was stabilized by forming multiple H-bonds within the binding pocket. At a 2.1 Å distance, the pyrazine ring’s amino group forms an H-bond with the hydroxyl group of Phe47. In addition, the hydroxyl and amine groups of Asn100 established H-bonds at a distance of 2.3 and 1.6 Å, respectively, with the hydroxyl groups of the tetrahydropentyl moiety of the compound. One new H-bond was formed during MD simulation between the OH group at fourth position of the same moiety and Asn100 (Figure 19D). The water bridge of the compound with Thr50 and Tyr106 was replaced by Ile53.

ZINC95589781 was stabilized in the binding pocket by forming two H-bonds. The amide group and carboxyl group of carboxyamide next to the thieno pyrimidine ring formed an H-bond with Phe47 and Tyr57 at a distance of 1.9 and 3.1 Å, respectively. This compound was also engaged in multiple hydrophobic interactions with Val49, Tyr85, Leu109, as well as water bridging with Asn100 (Figure 19E).

### 2.7. Molecular Mechanics/Generalized Born Surface Area

**Figure 20:**
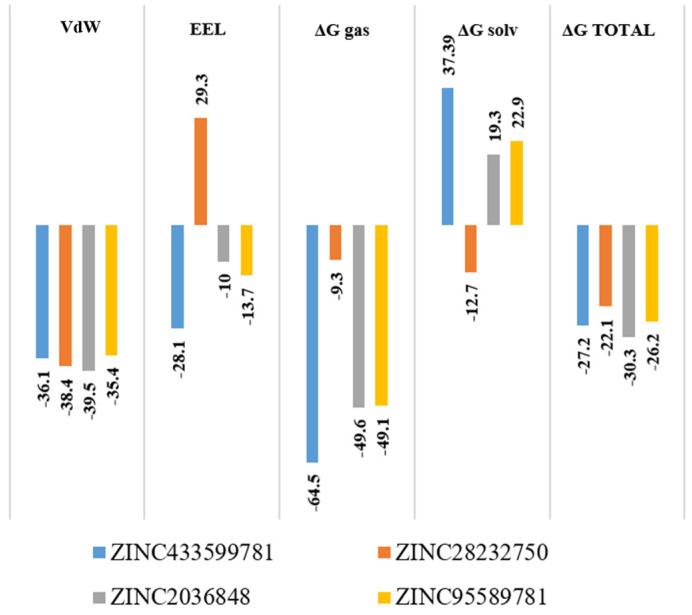
Predicted ΔG binding energy kcal/mol of the top four compounds by MM-GBSA.

In a 100 ns MD simulation study, the average interaction energy ranged from −30.3 to −22.1 kcal/mol, which indicates the favorable and stable complex formation. The total binding free energy was decomposed into various energy components to investigate the chief driving force in complex formation. It was noted that ΔEvdW varies between −35.1 and −39.5 kcal/mol, while ΔE_ele_ varies between −10 kcal/mol to −29.3 kcal/mol for all complexes (Figure 20). It suggests that the higher MM-GBSA values (ΔGbind) in the case of these complexes were mostly contributed to by the van der Waals and hydrophobic interactions. Among all screened compounds, ZINC2036848 binds more strongly, as it exhibited the most favorable values for ΔEvdW (−39.5 kcal/mol). The overall net polar contributions (ΔEele + ΔGpol) for ZINC433599781, ZINC28232750, ZINC2036848 and ZINC95589781 were found to be nearly close and unfavorable, being 8.9, 16.6, 9.3, and 9.2 kcal/mol respectively. However, for all complexes, the overall non-polar contributions (ΔEvdW + ΔGnp) were found to be −100.6 −47.7, −89.1, and −84.5 kcal/mol for ZINC433599781, ZINC28232750, ZINC2036848 and ZINC95589781, respectively. Conversely, the polar solvation energy (ΔGpol) opposed the complex formation for all the compounds except for ZINC28232750, which exhibited −12.7 kcal/mol. This suggests that the higher binding affinity for the complexes was due to more favorable non-polar components compared to the polar energy components.

### 2.8. Pharmacokinetics Analysis

Analysis revealed that the molecular weight (MW) of all the compounds except ZINC28232750 (FDA approved) was less than 500 Kdal, which suggests that all the compounds may easily be transported, diffused and absorbed inside the body. The number of H-bond donors ranged from two to five, and the number of H-bond acceptors was in the range from four to eight for all compounds except for ZINC28232750, which is in accordance with Lipinski’s rules of five. The bio availability score of all the hits was found to be 0.55, indicating good bioavailability of these compounds, excluding ZINC28232750. Transport processes, including membrane permeability and distribution to different tissues and organs, are closely predicted by LogP_0/w_. A general guide for good oral bioavailability (good permeability and solubility) is to have a moderate LogP_0/w_ (0 < log P < 3) [24]. For our compounds, the predicted values of LogP_0/w_ ranged from 1.6 to 3.6. In addition, all the compounds were confirmed to be blood–brain barrier [BBB] non-permeable, suggesting no expected neurological side effects. However, pharmacokinetics analysis indicated the low GI absorption of ZINC433599781, ZINC28232750 and ZINC2036848, suggesting the need for derivatization of these compounds to improve their ADME profile. An ADME profile of all the compounds is compiled in Table 4.

## 3. Materials and Methods

### 3.1. Protein Preparation

To date, more than 40 crystal structures of BRD9 have been deposited in the protein data bank (PDB) repository. In total, four different structures of BRD9, co-crystallized with BI7273, LP99, I-BRD9, and Indolizine comp-28, were retrieved. The selection was based on the optimal resolution (<2 Å) and IC_50_ (<500 nM) of the cognate ligand. The heteroatoms and crystal water (except the conserved ones) were eliminated, followed by the protonation at 300 K temperature with pH 7.0. The energy minimization and subsequent application of partial charges was done using AMBER 10: EHT force field in MOE 2018 [22].

### 3.2. Pharmacophore-Based Virtual Screening

#### 3.2.1. Dataset Preparation

Three data sets were prepared.

1. Test Set: Actives (IC_50_ < 500 nM) and inactives (IC_50_ > 500 nM) of BRD9, and actives of BRD4 (Supplementary Information Figures S1, S2, and S3, respectively).
2. Screening dataset: Four subsets; Predicted BRD9, FDA-approved, In-trial, and Epigenetic were downloaded from ZINC database.
3. Decoys: The ZINC database was used to extract the decoy dataset, which contains compounds that share the same physical properties as BRD9 actives but differ in topology. The final decoy database was composed of 12, 991 entries.

ChemDraw Bioultra 14.0 [25] was used for drawing the 2D structures of active and inactive compounds. All ligands were imported to MOE 2018, where partial charges were assigned, followed by energy minimization according to the MMFF94x force-field [26] to remove steric clashes.

#### 3.2.2. Pharmacophore Modeling

For the generation of a structure-based pharmacophore model, the essential step is to explore the complementary chemical features and crucial residue of the binding site [27,28]. The four crystal structures of BRD9 reported in Section 2.1 were selected to map the pharmacophoric features (Table 5). All the four crystal structures were aligned by the backbones of amino acids using MOE 2018. The molecular visualization and protein–ligand interaction profile (PLIF) module evaluated the identification of binding mode and inter-molecular interaction patterns. The unified annotation scheme was used to generate pharmacophore models [29].

The initially developed pharmacophore model was first optimized to enhance the model’s predictive performance. The validation was carried out by screening the model against training and decoy datasets to ensure the capabilities of the generated pharmacophore model to differentiate between true and false positive. The model was further refined by changing the radii of some pharmacophoric features and by applying an exclusion volume.

#### 3.2.3. Statistical Validation

The capability of the pharmacophore model to recognize the maximum number of active hits and reject of inactive ones reflects its efficacy. Therefore, the quality of the developed 3D pharmacophore model was assessed by the two fundamental metrics, i.e., sensitivity and specificity. Further, the correlation between sensitivity and specificity was determined by the receiver operating characteristic (ROC) curve [30,31].

#### 3.2.4. Virtual Screening

The validated structure-based pharmacophore model was used as a 3D query for pharmacophore-based virtual screening of commercially available diverse subsets of the ZINC database (screening dataset mentioned in Section 3.2.1). Database searching was carried out using MOE software’s best per conformation generation tool. The molecules in the databases that optimally satisfied the pharmacophore model’s applied features were retained as hits and further subjected to molecular docking.

### 3.3. Docking Simulation

#### 3.3.1. Benchmarking of Docking Software

**Figure 21:**
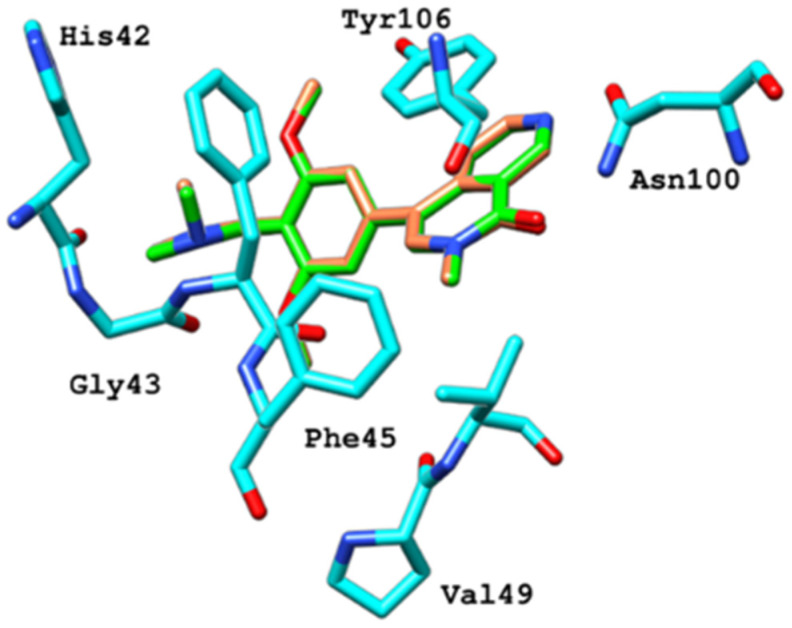
Re-docked conformation of a potent BRD9 inhibitor (BI-7273) inside the active site of BRD9 (PDB ID: 5EU1): crystallographic pose and re-dock pose (orange) using MOE 2018 [22].

To validate the reproducibility of the docking software, a co-crystallized ligand (BI-7273) of BRD9 was extracted, prepared, energetically minimized (mentioned in Section 3.2.1), and redocked to the binding site by using the induced fit method from the Dock module implemented in MOE 2018. The Triangle Matcher placement method and the London dG scoring function was used to generate the docking poses of the ligand and their binding free energy. However, the generated poses were further refined using the GBVI/WSA dG scoring function [32]. The results shown in Figure 21 indicate the very good superposition of the co-crystalized and re-docked pose of BI-7273 in the binding pocket, with an RMSD of 0.12 Å. This value is lower than the standard acceptable level of 2.0 Å, thus the tested docking protocol was used to perform molecular docking of the obtained hits from the subsequent step [33].

#### 3.3.2. Docking-Based Virtual Screening

To predict the orientation into the binding cavity of the targeted protein and further filter the compounds based on relative binding affinities, the virtual hits from the pharmacophore-based screening were docked into the acetyl binding site of the receptor. The docking was performed utilizing the same protocol mentioned in Section 3.3.1.

#### 3.3.3. Post Docking Assessment

Docking was followed by protein–ligand interaction profile analysis and molecular visualization of all ligand–receptor complexes’ top-ranked pose (lowest binding energy). The selection was based on the interaction of compounds with crucial and selective residues of the target protein.

Furthermore, to confirm the selectivity of the shortlisted compounds with BRD9 over other isoforms, redocking was performed against BRD7 (PDB: ID 5MQ1) [34] by using the same docking protocol as mentioned earlier. Finally, only those compounds which selectively interacted with the crucial and selective residues of the binding pocket of BRD9 were subjected to atomistic level simulation studies.

### 3.4. Molecular Dynamic Simulation

The shortlisted hits (ZINC433599781, ZINC28232750, ZINC2036848, ZINC95589781) from molecular docking and the reference compound were subjected to MD simulation to capture their dynamic behavior at the atom level while gaining further insights regarding the nature of interactions for a given time.

To execute MD simulations on all the prepared complexes, the AMBER 18 package was used, using the graphics processing unit (GPU)-accelerated version of partial mesh Ewald molecular dynamics (PMEMD) simulations [35].

The LEaP module of the AMBER18 package was used to add all the missing hydrogen atoms [36]. The appropriate parameters for the protein atoms were applied using the standard Amberff14SB force field [37], while the small molecules were parameterized with the general amber force field (GAFF) using the Antechamber program [38]. It was followed by the placement of the generated complexes in a periodic rectangular box filled with three-point transferable intermolecular potential (TIP3P) water molecules [39] with a distance cutoff of 8 Å in the vicinity of the complex. Further, chloride ions were added to maintain the neutrality of the systems. The bad contacts and steric clashes were removed by subjecting the complexes to the initial cycle of (2500 steps) of steepest descent minimization followed by energy minimization using the conjugate gradient algorithms (comprising 5000 steps) [40]. All systems were then gradually heated from 0 to 300 °K. Minimization was followed by equilibration, which was carried out at 300 °K to attain a stable system. Subsequently, the NPT equilibration for the unrestrained system was carried out at a constant pressure and temperature (1 atm and 300 °K, respectively).

The energy-minimized and well-equilibrated systems were submitted for a 100 ns production run [41]. During the simulations, periodic boundary conditions were applied at a constant temperature and pressure. Long-range electrostatic interactions were evaluated by the particle-mesh Ewald (PME) method [42]. For all simulations, a 10.0 Å cutoff was set for non-bonded interactions. The SHAKE algorithm was used to constrain the bond lengths of all atoms, including hydrogen atoms [43]. Simulation results were elucidated based on statistical parameters including root mean square deviation (RMSD), root mean square fluctuation (RMSF), the radius of gyration (RoG), and molecular interactions. Trajectory analysis was carried out by using the CPPTRAJ [44], and visualization was performed by VMD [45].

### 3.5. Molecular Mechanics/Generalized Born Surface Area

Molecular mechanics/generalized born surface area (MMGBSA) was used to calculate protein and small-molecule complexes. It has been effectively used to refine the results of virtual screening, docking, and to confirm the experimental findings [46,47].

We applied this method for the most-equilibrated MD trajectories. To calculate binding free energies, a python script, MMGBSA.py, was used.

In particular, the energy is calculated for the complex, the ligand, and the protein, and their energies are estimated with the generalized born implicit solvent model by the following equation:

ΔG_bind_ = G_Complex_ − (G_ligand_ + G_receptor_) (3)

where the energy term [G] is estimated as:

ΔG_gas_ = E_vdw_ + E_ele_ + E_int_ (4)

ΔG_sol_ = GB + G_SA_ (5)

E_vdw_, E_ele_, E_int_ as the van der Waals forces, electrostatic interactions, and internal energy (bond, angle, and dihedral energies), respectively.

ΔGB is the electrostatic contribution to the solvation free energy calculated by the generalized Boltzmann method and GSA is a nonpolar contribution to the solvation free energy calculated by SASA (solvent accessible surface area).

### 3.6. Pharmacokinetic Properties Analysis

Swiss ADME [48], an online software, was used to predict pharmacokinetic properties of the hit compounds. Absorption, distribution, metabolism, and excretion parameters were mainly examined.

## 4. Conclusions

Bromodomain-containing protein 9 has piqued the interest of drug developers as a biomarker and therapeutic target for different types of cancer and metabolic disorders. Given the medicinal relevance, the discovery and development of selective inhibitors for BRD9 have been under continuous efforts. In the present study, we identified small molecules that selectively target BRD9. In this regard, the structure-based pharmacophore model was designed to screen four subsets of the ZINC database to obtain novel leads. The leads were further refined by docking and focusing on its interaction with selective residues of BRD9 binding pocket. Four compounds (ZINC433599781, ZINC282232750, ZINC2036848, and ZINC9558978) were identified. Further, the determination of stability was performed via molecular dynamics simulation. The results of RMSD, RMSF, and RoG confirm the stability and compactness of all the complexes. Hydrogen bonding, hydrophobic interactions, π–π interactions and water bridges with conserved and selective residues such as His42, Gly43, Phe44, Phe45, Asn100 and Tyr106 of BRD9 contribute to the stability and binding of all the compounds. Moreover, the binding energy for the formation of all the complexes was calculated using MM-GBSA, which was found to be negative and represents the strong binding of the ligand with the BRD9. Additionally, the results obtained from docking (−7.1 kcal/mol), MM-GBSA (−30.2 Kcal/mol) and post-MD analysis demonstrated movement of the molecule in the center of the cavity. Moreover, BRD9–ZINC2036848 complex interactions were reasserted during 100 ns MD simulation, which indicated that ZINC2036848 might be used as a potent antagonist towards BRD9 in cancer therapeutics.

In view of the fact that so far no treatment has been devised for BRD9 mediated disorders, the data presented here could be helpful in the future for in vitro and in vivo studies and could be used for further expansion in the chemical space.

## Figures and Tables

**Table 1:** Pharmacophore hit-rates of different data sets.

Dataset	No. of Compounds	Hit Rate
Actives	17	13 (76.4)
Inactives	8	1 (12.5)
BRD4 Inhibitors	25	0 (0.0)
Decoys	12,991	154 (1.8)

**Table 2:** Pharmacophore-based virtual screening hits obtained from each subset of the ZINC database.

Database	Total Compounds	Lead Compounds
Predicted-BRD9 Compounds	25,532	596
FDA approved Drugs	1466	11
In-Trials Compounds	6799	101
Epigenetic Compounds	471	6

**Table 3:** Summary of the four shortlisted compounds with binding energy kcal/mol.

S.no	Name	Score	Structure
1	ZINC433599781	−7.3	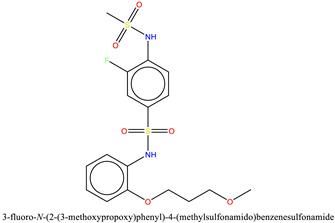
2	ZINC28232750 (Valstar)	−7.5	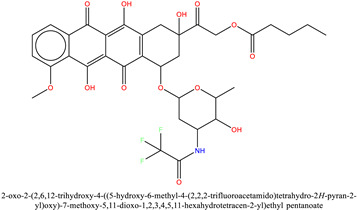
3	ZINC2036848 (Riboflavin)	−7.1	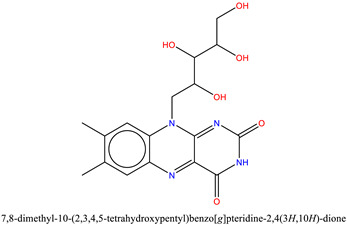
4	ZINC95589781	−6.8	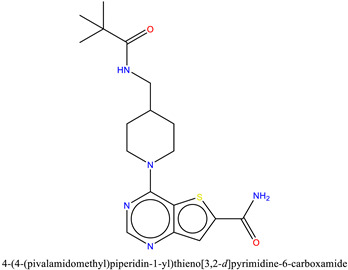

**Table 4:** ADME analysis of top four hits using Swiss ADME.

Compound ID	ZINC433599781	ZINC28232750	ZINC2036848	ZINC95589781
MW (g/mol)	423.4	723.6	376.6	375.4
LogP_0/w_	2.25	3.68	1.63	2.46
Log (ESOL)	Soluble	Moderately Soluble	Very soluble	Soluble
GI absorption	Low	Low	Low	High
Bioavailability	0.55	0.17	0.55	0.55
BBB	No	No	No	No
H-bond acceptor	7	16	8	4
H-bond donor	2	5	5	2
Lipinski’s Rule of Five	Yes	Yes	Yes	Yes

MW = Molecular weight; LogP_0/w_ = Octanol Partition Coefficient; LogS = Aqueous Solubility; GI = Gastrointestinal Absorption.

**Table 5:** List of BRD9 inhibitors that have been used in the development of the pharmacophore model, along with their PDB IDs, resolutions and IC_50_/K_d_ (nM) values.

PDB ID	Resolution	Cognate Ligand	IC_50_ (nM)
4UIW	1.7 Å	I-BRD9	50
5IGN	1.7 Å	LP99	325
5EU1	1.6 Å	BI-7273	19
5E9V	1.8 Å	Indolizine com28	68

## Data Availability

Not applicable.

## Supplementary Materials

The following supporting information can be downloaded at: https://www.mdpi.com/article/10.3390/ijms232113513/s1.
