# Supplementary material for: Identification of Selective BRD9 Inhibitor via Integrated Computational Approach
Source: Int J Mol Sci. 2022 Nov 4;23(21):13513. doi: 10.3390/ijms232113513 (PMC9655433; doi:10.3390/ijms232113513)
Supplement: Supplementary file 1 [file ijms-23-13513-s001.zip › Figure S3.pdf]

Figure S3. **List of BRD4 active compounds with IC_{50} (nM values)**

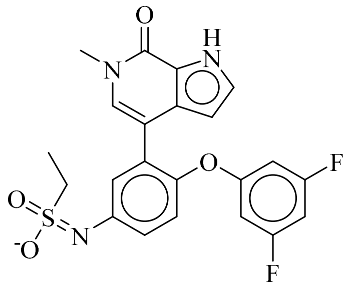

1 (NA)

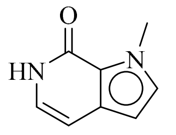

2 ($1x10^{5}$ nM)

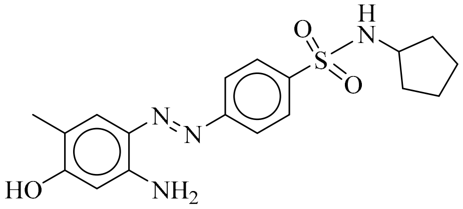

3 (49 nM)

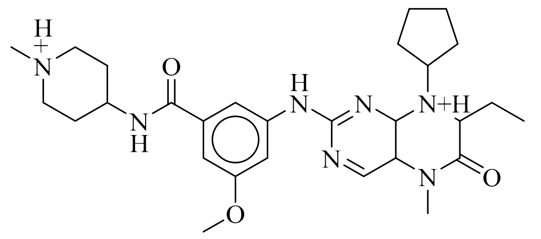

4 (51 nM)

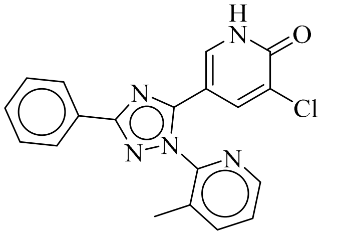

5 ($47x10^{2}$ nM)

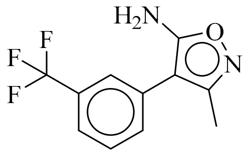

6 ($81x10^{3}$ nM)

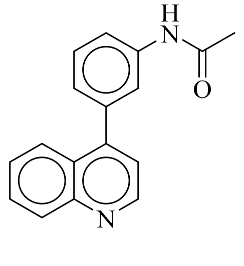

7 ($25x10^{4}$ nM)

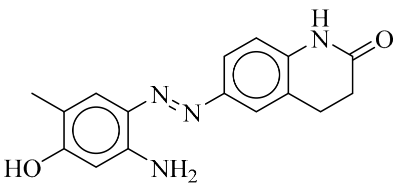

8 (27 nM)

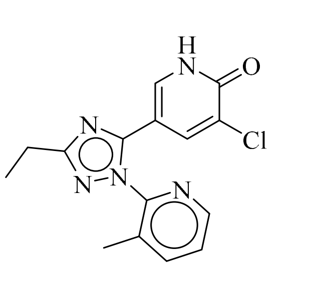

9 ($1x10^{5}$ nM)

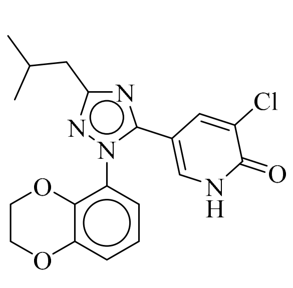

10 ($77x10^{3}$ nM)

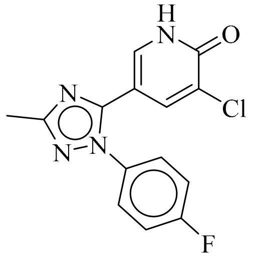

11 ($1x10^{5}$ nM)

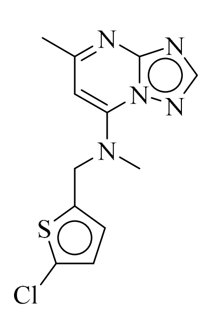 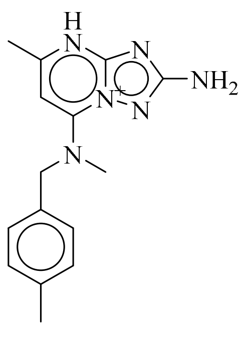 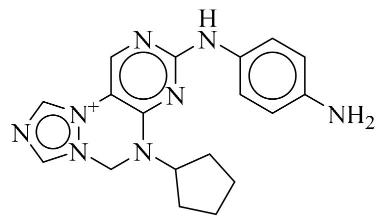

12 ($24x10^3$ nM) **13 ($66x10^3$ nM)** **14 (79 nM)**

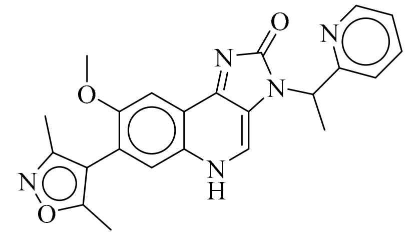 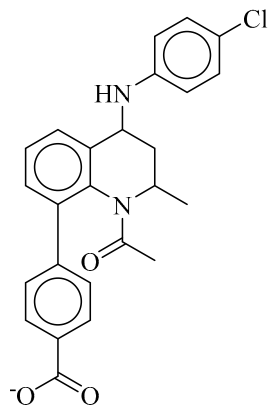 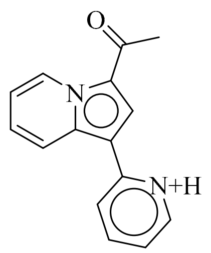

15 ($79x10^1$ nM) **16 (42 nM)** **17 ($2x10^3$ nM)**

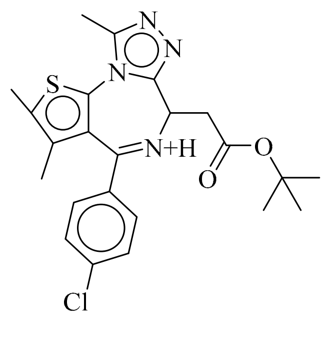 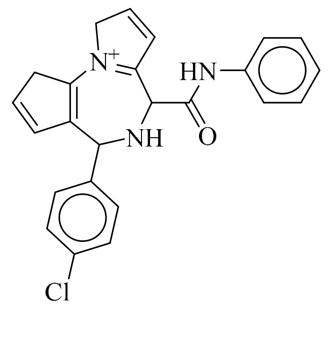 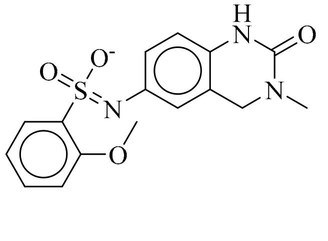

18 (66 nM) **19 (112 nM)** **20 (220 nM)**

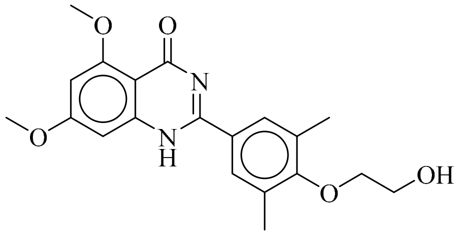

21 (87x10^3 nM)

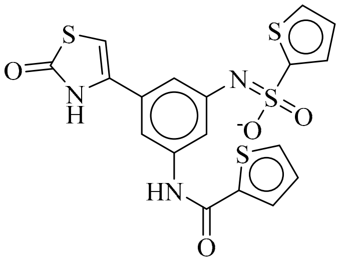

22 (230 nM)

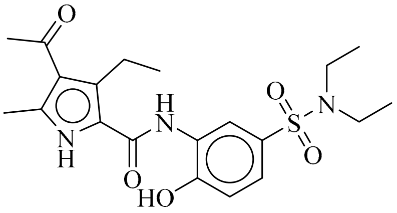

23 (17x10^3 nM)

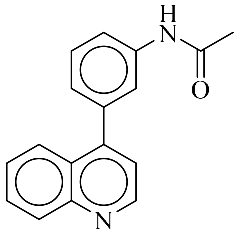

24 (25x10^4 nM)

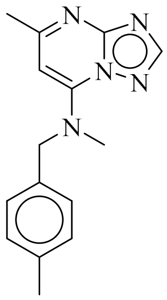

25 (25x10^4 nM)
