# Supplementary figures and images for: Identification of Selective BRD9 Inhibitor via Integrated Computational Approach
Source: Int J Mol Sci. 2022 Nov 4;23(21):13513. doi: 10.3390/ijms232113513 (PMC9655433; doi:10.3390/ijms232113513)

**Figure S1.** List of Active compounds of BRD9 with IC<sub>50</sub> (nM) values

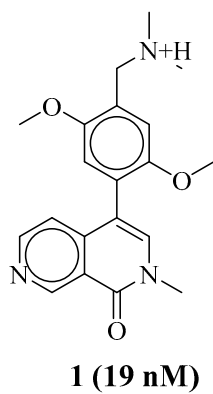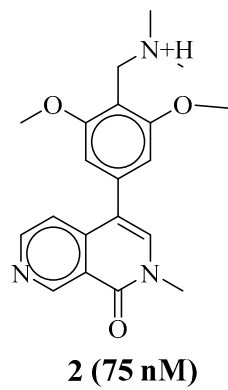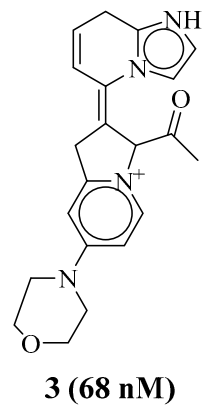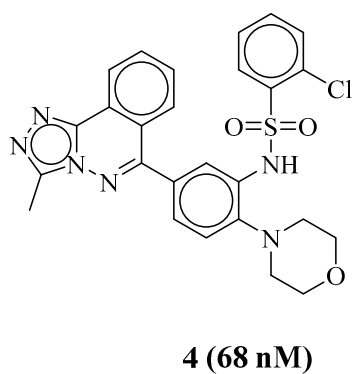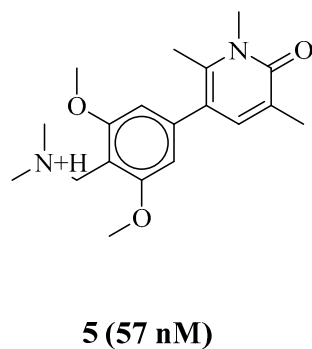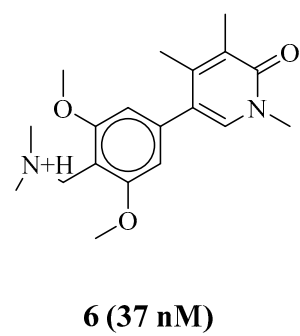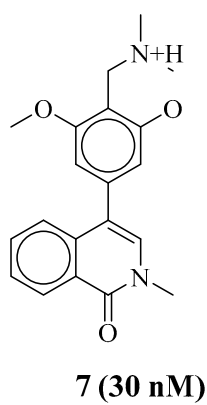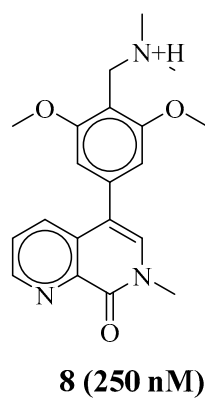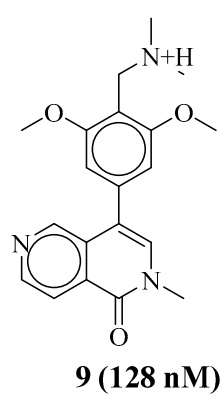

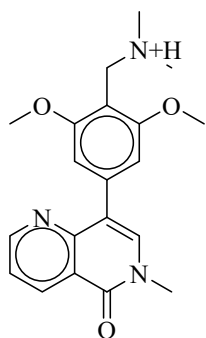

**10 (134 nM)**

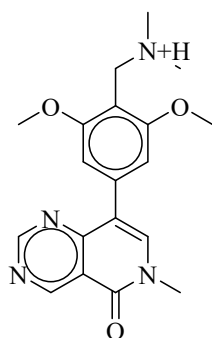

**11 (37 nM)**

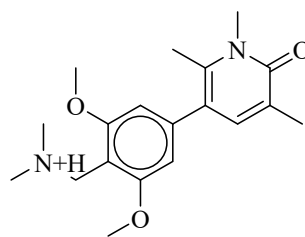

**12 (134 nM)**

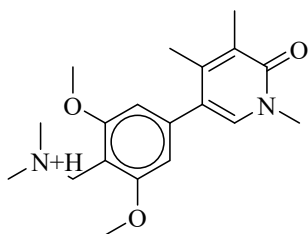

**13 (507 nM)**

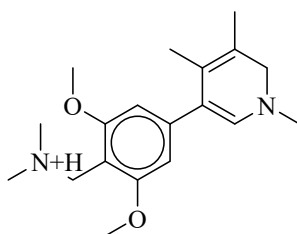

**14 (134 nM)**

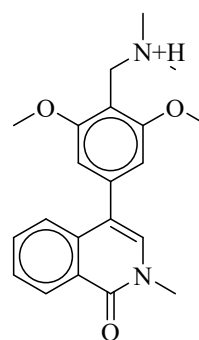

**15 (54 nM)**

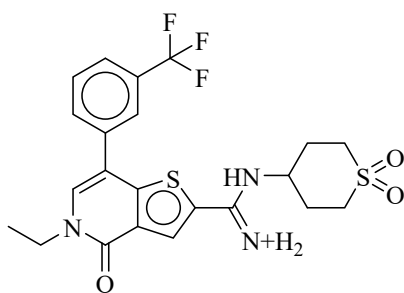

**16 (50 nM)**

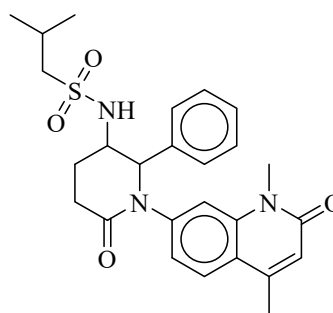

**17 (325 nM)**

Supplement: Supplementary file 1 [file ijms-23-13513-s001.zip › Figure S1.pdf]

**Figure S2. List of inactives of BRD9**

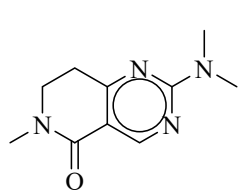

**1 (489x10<sup>2</sup> nM)**

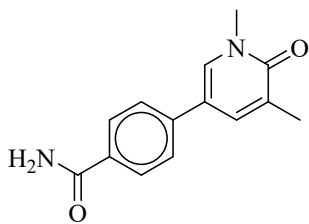

**2 (9398 nM)**

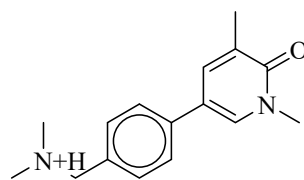

**3 (1147 nM)**

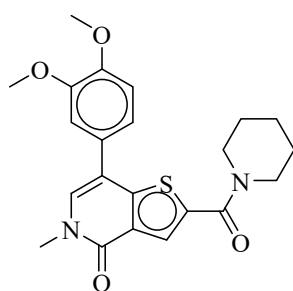

**4 (3980 nM)**

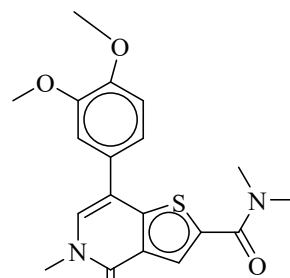

**5 (5010 nM)**

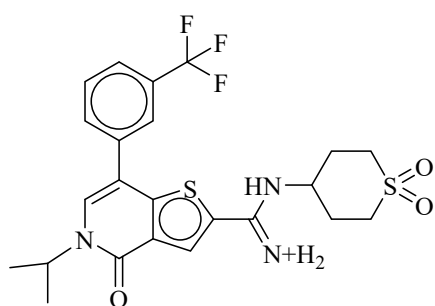

**6 (3160 nM)**

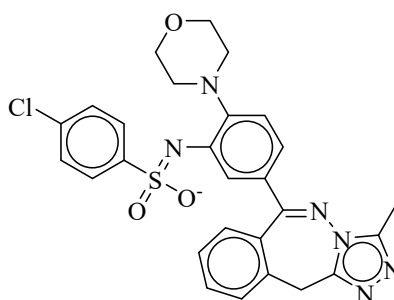

**7 (10<sup>3</sup> nM)**

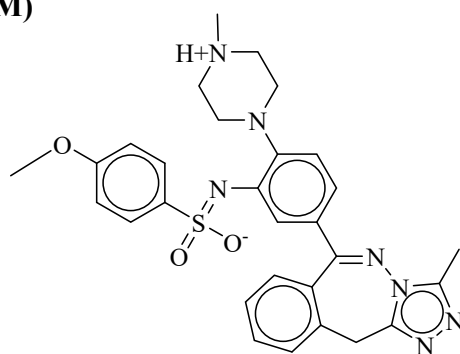

**8 (630 nM)**

Supplement: Supplementary file 1 [file ijms-23-13513-s001.zip › Figure S2.pdf]
